# Haploinsufficiency for p190B RhoGAP inhibits MMTV-Neu tumor progression

**DOI:** 10.1186/bcr2352

**Published:** 2009-08-24

**Authors:** Brandy M Heckman-Stoddard, Tracy Vargo-Gogola, Peter R McHenry, Vivian Jiang, Matthew P Herrick, Susan G Hilsenbeck, Jeffrey Settleman, Jeffrey M Rosen

**Affiliations:** 1 Department of Molecular and Cellular Biology, Baylor College of Medicine, One Baylor Plaza, Houston, TX 77030, USA; 2 Cancer Prevention Fellowship Program, Center for Cancer Training, National Cancer Institute, 6120 Executive Boulevard, Bethesda, MD 20852, USA; 3 Department of Biochemistry and Molecular Biology, Indiana University School of Medicine, 1234 Notre Dame Avenue, South Bend, IN 46617, USA; 4 Lester and Sue Smith Breast Center and Department of Medicine, Baylor College of Medicine, One Baylor Plaza, Houston, TX 77030, USA; 5 Massachusetts General Hospital Cancer Center and Harvard Medical School, 13th Street, Charlestown, MA 02129, USA

## Abstract

**Introduction:**

Rho signaling regulates key cellular processes including proliferation, survival, and migration, and it has been implicated in the development of many types of cancer including breast cancer. P190B Rho GTPase activating protein (RhoGAP) functions as a major inhibitor of the Rho GTPases. P190B is required for mammary gland morphogenesis, and overexpression of p190B in the mammary gland induces hyperplastic lesions. Hence, we hypothesized that p190B may play a pivotal role in mammary tumorigenesis.

**Methods:**

To investigate the effects of loss of p190B function on mammary tumor progression, p190B heterozygous mice were crossed with an MMTV-Neu breast cancer model. Effects of p190B deficiency on tumor latency, multiplicity, growth, preneoplastic progression and metastasis were evaluated. To investigate potential differences in tumor angiogenesis between the two groups, immunohistochemistry to detect von Willebrand factor was performed and quantified. To examine gene expression of potential mediators of the angiogenic switch, an angiogenesis PCR array was utilized and results were confirmed using immunohistochemistry. Finally, reciprocal transplantation of tumor fragments was performed to determine the impact of stromal deficiency of p190B on tumor angiogenesis.

**Results:**

P190B deficiency reduced tumor penetrance (53% of p190B^+/-^Neu mice vs. 100% of p190B^+/+^Neu mice formed tumors) and markedly delayed tumor onset by an average of 46 weeks. Tumor multiplicity was also decreased, but an increase in the number of preneoplastic lesions was detected indicating that p190B deficiency inhibited preneoplastic progression. Angiogenesis was decreased in the p190B heterozygous tumors, and expression of a potent angiogenic inhibitor, thrombospondin-1, was elevated in p190B^+/-^Neu mammary glands. Transplantation of p190B^+/-^Neu tumor fragments into wild-type recipients restored tumor angiogenesis. Strikingly, p190B^+/+^Neu tumor fragments were unable to grow when transplanted into p190B^+/-^Neu recipients.

**Conclusions:**

These data suggest that p190B haploinsufficiency in the epithelium inhibits MMTV-Neu tumor initiation. Furthermore, p190B deficiency in the vasculature is responsible, in part, for the inhibition of MMTV-Neu tumor progression.

## Introduction

Small GTPases of the Rho family act as nodes of signal transduction, integrating extracellular signals to affect actin cytoskeletal organization, cell adhesion, polarity, proliferation and migration, which are all important processes that become deregulated during cancer progression. Rho proteins are molecular switches that cycle between an active GTP-bound state and an inactive GDP-bound state, and their activities are spatially and temporally regulated. Three classes of proteins control Rho activity, including guanine nucleotide exchange factors that activate the switch, GTPase activating proteins (GAPs) that inactivate the switch, and guanine nucleotide dissociation inhibitors that prevent the exchange of GDP for GTP on the endogenous RhoGTPases [1-3]. Several studies have shown overexpression of Rho family members in human breast cancer samples by immunohistochemistry, by mutational analysis, or by RNA expression profiling [4-7]. The role of Rho signaling in breast cancer, however, is not well understood.

To elucidate the contribution of the Rho signaling pathway to the growth and progression of breast cancer, we focused on understanding the role of p190B RhoGAP, which has been shown previously to play an important role in normal mammary gland development [8,9]. P190B is a member of the RhoGAP family, which acts as negative regulator of Rho activity. The GAP domain of p190B demonstrated activity against Rho, Rac and Cdc42 [10], and more recently p190B was shown to directly bind Rnd3, Rac1 and RhoA [11]. Homozygous deletion of p190B results in central nervous system and lung defects, leading to perinatal lethality [12]. P190B-deficient embryos and cells are smaller than their wild-type counterparts, due in part to impaired insulin-like growth factor signaling. Importantly, the p190B and insulin-like growth factor signaling pathways have been shown to directly interact through Rho kinase (ROK) phosphorylation of insulin receptor substrate proteins and to be critically involved in regulating both cell growth and differentiation [12,13].

P190B is highly expressed throughout embryonic and virgin mammary gland development, with expression decreasing during late pregnancy and remaining low during lactation [14,15]. P190B homozygous-deficient mammary glands fail to undergo ductal morphogenesis. Moreover, loss of one allele of p190B transiently delays ductal morphogenesis and results in decreased proliferation within the terminal end buds, which drive ductal penetration into the fat pad [8]. Overexpression of p190B during virgin development disrupts terminal end bud morphogenesis, increases side branching, and delays ductal elongation, while overexpression during pregnancy results in hyperplastic lesions [9].

To elucidate the role of p190B in mammary tumor progression, we crossed p190B heterozygous mice with a mouse mammary tumor virus (MMTV)-Neu mouse model of breast cancer and examined multiple stages of tumor progression. Neu/ErbB2 is a member of the epidermal growth factor receptor family that is amplified and overexpressed in 20 to 30% of human breast cancers and correlates with poor prognosis [16]. Several mouse models have been generated to examine mechanisms involved in ErbB2-mediated mammary tumor progression [17]. For these studies, we chose to use the MMTV-Neu model that overexpresses a wild-type *Neu *proto-oncogene [18]. MMTV-Neu female mice develop preneoplastic lesions, some of which progress to adenocarcinomas, with a median onset of 7 months of age. Approximately 70 to 80% of tumor-bearing mice develop metastasic disease [18]. GAPs and related G proteins are reportedly upregulated in these tumors, suggesting that Rho signaling is important for MMTV-Neu tumor formation and progression [19].

Using this approach, we demonstrate that p190B plays a critical role in MMTV-Neu-induced tumorigenesis. Strikingly, loss of one allele of p190B decreased tumor penetrance, delayed tumor onset, and reduced metastasis. Furthermore, the progression of preneoplastic lesions was inhibited by the loss of p190B, and p190B heterozygous tumors have a reduced vascular network, thus implicating p190B in tumor angiogenesis. Reciprocal transplantation of tumor fragments confirmed that p190B expression in the stroma plays an important role in tumor angiogenesis. The effects of p190B loss on angiogenesis may be due in part to increased expression of a potent anti-angiogenic factor, thrombospondin-1 (TBS-1). The reduction in angiogenesis may contribute to the inhibition of metastasis.

## Materials and methods

## Mice strains and tumor analysis

P190B null mice [12] were backcrossed to FVB for four generations. P190B^+/- ^(FVB4) mice were then crossed to MMTV-Neu transgenic mice, a fifth cross to FVB, to yield control and experimental animals. Mice were fed a conventional diet *ad libitum *and were maintained at 21 to 22°C with a 12-hour light and 12-hour dark cycle.

Animal protocols were approved by the Animal Care and Use Committee of Baylor College of Medicine, and were conducted in accordance with the provisions of the Guide for the Care and Use of Laboratory Animals and the Animal Welfare Act.

Mice were monitored weekly by palpation for tumor induction. Individual tumor size and location was recorded. Twenty p190B^+/+^/MMTV-Neu littermates and 15 p190B^+/-^/MMTV-Neu littermates were analyzed, and Kaplan-Meier analysis was performed to determine tumor-free survival. Log-rank test analysis was performed to determine whether differences between the two groups were statistically significant.

## Tissue preparation

Heterozygous females (p190B^+/- ^FVB5:C57Bl/6) were mated with heterozygous males (MMTV-Neu FVB), with detection of tumors taking place in the F1 generation. Mice at tumor burden (1.0 mm^3^) were given an intraperitoneal injection of bromodeoxyuridine (100 mg/kg). Two hours later, the tumors, mammary glands, and lungs were dissected and fixed for 2 hours in 4% paraformaldehyde. Tumor and lung specimens were dehydrated and embedded in paraffin. Gland specimens were defatted with acetone and stained with Neutral Red overnight, followed by clearing with xylene [20]. Whole-mount pictures were taken using a stereomicroscope (Leica, Bannockburn, IL, USA; Model M165 C). After imaging, whole-mounted mammary glands were paraffin embedded. Five-micrometer serial sections were cut into the frontal plane for subsequent histological and immunostaining analysis.

## Preparation of tumor lysates and western blotting

Protein lysates were prepared as previously described [9]. Protein concentrations in the tissue extracts, p190B^+/+^Neu (n = 5) or p190B^+/-^Neu (n = 5), were determined using the BCA Protein Quantitation Assay (Pierce, Rockford, IL, USA). Extracts were pooled (20 μg of each), electrophoresed on 6% or 12% SDS-PAGE gels, and transferred to polyvinylidene fluoride (PVDF) membrane (Millipore, Bedford, MA, USA). Membranes were blocked in 5% milk/Tris-buffered saline Tween-20 followed by incubation with p190B 1:1,000 antibody (BD Bioscience, San Jose, CA, USA), ErbB2/Neu 1:1,000 (Neomarkers, Fremont, CA, USA), ErbB3 1:1,000 (Santa Cruz, Santa Cruz, CA, USA), ERK 1:1,000 (Cell Signaling, Danvers, MA, USA), phospho-p21-activated kinase (PAK)1/2 1:1,000 (Cell Signaling), and β-actin 1:5,000 (Sigma, St Louis MO, USA) in 5% milk/Tris-buffered saline Tween-20. Other western blot assays were completed and developed as previously described [9].

## Immunohistochemistry and quantification of staining

Immunohistochemistry was carried out as previously reported [9] using cleaved caspase 3 (9961; Cell Signaling), 1:200 in 5% BSA, 0.5% blocking buffer; biotin-conjugated bromodeoxyuridine (550803; BD Pharmingen), 1:10 in 5% BSA, 0.5% blocking buffer; and TBS-1 (NeoMarkers), 1:75 in MOM block (Vector Laboratories, Burlingame, CA, USA). Immunofluorescence for von Willebrand factor (VWF) was performed according to the manufacturer's protocol using anti-VWF (DAKO, Glostrup Denmark), 1:200 in 5% BSA, 0.5% blocking buffer.

Two independent observers blinded to the experimental groups assessed the peritumoral vascular density by counting the number of vessels within three 100× fields from each sample. Quantification of TBS-1 staining was performed by counting the number of TBS-1-positive cells in five fields from one gland from each of five mice per genotype.

## RNA extraction and quantitative real-time PCR

RNA was isolated from briefly digested (collagenase 2 mg/ml for 1 hour) mammary glands to remove adipose tissue of p190B^+/+^Neu and p190B^+/-^Neu adult virgin mice according to the manufacturer's protocol (SABiosciences, Frederick, MD, USA). Briefly, Trizol (Invitrogen, Carlsbad, CA, USA) solution was used to according to the manufacturer's protocol followed by an additional purification step using the RNA easy kit (Qiagen, Germantown, MD, USA). The expression of mRNAs involved in the angiogenesis pathway was determined using real-time PCR and a SuperArray Profiler PCR array (SABiosciences, Frederick, MD, USA).

For quantification, cDNA was synthesized using 1 μg RNA in a reverse transcription reaction (SABiosciences). cDNA was amplified using the SYBR Green qPCR Master Mix (SABiosciences) with a real-time PCR Applied Biosystems 7500 thermocycler (Applied Biosystems Inc, Foster City, CA, USA). Samples were run in triplicate.

Manufacturer-supplied primer pairs were used to measure mRNAs expressed in the angiogenesis pathway (PAMM-024; SABiosciences) as described in the manufacturer's protocol. The mRNA expression levels were normalized to the expression level of 4/5 housekeeping genes included in the array, excluding β-actin. The positive threshold was determined based on negative controls as described in the manufacturer's protocol. The calculations for determining relative gene expression were made using the cycle threshold method and web-based PCR array data analysis (SABiosciences).

## GTPase activity assays

Activities of Rho family GTPases were measured using luminescent (RhoA and Rac1) or colorimetric (Cdc42) G-LISA assays (Cytoskeleton, Denver, CO, USA) according to the manufacturer's instructions. Frozen tumors were pulverized using a mortar and pestle, and were homogenized in lysis buffer containing protease inhibitors (Cytoskeleton) using a needle and syringe. The lysates were centrifuged for 2 minutes at 4°C and 14,000 × *g*, and the clarified lysates were aliquoted, snap-frozen in liquid nitrogen, and stored at -80°C.

The protein concentration was determined using the protein assay reagent provided. Immediately before the assays, the lysates were thawed and the protein concentrations were equalized (0.75 mg/ml for RhoA and Rac1, 2.0 mg/ml for Cdc42) using lysis buffer. For the luminescent assays, SuperSignal West Dura chemiluminescent substrate (Thermo Scientific, Rockford, IL, USA) was substituted for the kit substrate, and a Kodak Gel Logic 1500 digital imaging system and Kodak 1D software (Carestream Health, New Haven, CT, USA) were used to determine the luminescent intensity. For the colorimetric assay, a SpectraMax Plus 384 spectrophotometer and SoftMax Pro software (Molecular Devices, Sunnyvale, CA, USA) were used to determine the absorbance. Means were compared using a *t *test in GraphPad Prism software (GraphPad Software, Inc., La Jolla, CA, USA).

## Mammary tumor tissue transplantation

Three week-old SCID/Beige mice (Charles River, Germantown, MD, USA) were employed as hosts for transplantation. Both of the Number 4 inguinal mammary glands were cleared of endogenous epithelium. A single tumor piece (1 mm^3 ^in size) was implanted into an incision made in the cleared fat pad of the recipient gland. Mice were monitored weekly by palpation for tumor induction. The individual tumor size was recorded. Mice were euthanized when tumors reached 10 mm^3 ^in size and were analyzed as described above. For these studies, fragments from five tumors per genotype were each transplanted into 40 recipients. A Fisher's exact test was used to evaluate whether there was a statistically significant difference in transplantation take rates between the two groups.

For reciprocal transplants, 5-week old p190B^+/+^Neu mice and p190B^+/-^Neu mice were employed as hosts for transplantations. Both of the Number 4 glands were cleared of endogenous epithelium. A single tumor piece (1 mm^3 ^in size) was implanted into an incision made in the cleared fat pads of the recipient gland. Mice were monitored weekly by palpation for tumor induction, and the individual tumor size was recorded. Mice were euthanized when the total tumor burden reached 15 mm^3 ^in size and were analyzed as described above. For these studies, fragments from three tumors per genotype were transplanted into both Number 4 cleared glands of three recipient mice.

## Results

### Haploinsufficiency for p190B inhibits MMTV-Neu tumorigenesis

To examine whether p190B haploinsufficiency affects mammary tumorigenesis, we first backcrossed p190B heterozygous mice (C57Bl/6) into the FVB background to circumvent effects of strain-specific modifiers present in the C57Bl/6 background that have been reported to delay mammary tumorigenesis [21]. We previously reported that p190B heterozygosity resulted in a transient delay in mammary gland development between 4 and 6 weeks of age, but that subsequent mammary gland development and function was unaffected [8]. P190B heterozygous mice (C57Bl/6/FVB5) were then crossed with MMTV-Neu transgenic mice [18] to ascertain whether haploinsufficiency of p190B would have any effect on tumor initiation, progression and/or metastasis. The virgin offspring from these crosses were monitored by weekly palpation beginning at 3 months of age and were followed for 18 months.

**Figure 1:**
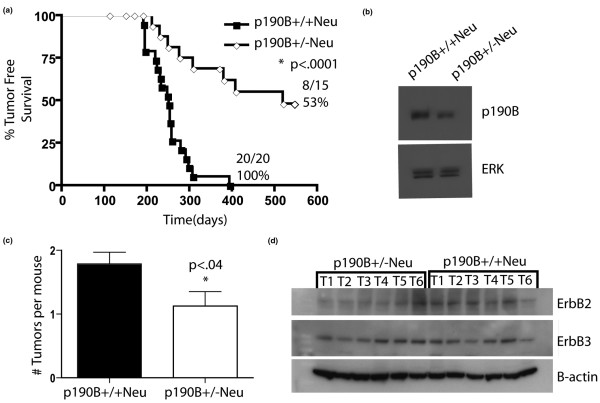
Haploinsufficiency for p190B delays MMTV-Neu tumor onset and causes a decrease in tumor multiplicity. **(a) **Kaplan-Meier tumor-free survival curve of p190B^+/+^Neu mice and p190B^+/-^Neu mice. The median time of tumor-free survival was 251 days and 591 days, respectively. Log-rank test analysis showed a significant difference between the two groups (*P *< 0.0001). **(b) **Immunoblotting for p190B and ERK in pooled tumor tissue from five tumors of indicated genotypes. **(c) **Tumor multiplicity for p190B^+/+^Neu mice and for p190B^+/-^Neu mice (1.8 and 1.2 tumors per mouse, respectively). Student's *t *test showed a significant decrease (*P *< 0.04). **(d) **Immunoblotting for ErbB2, ErbB3, and β-actin in six individual tumors from the indicated genotypes. Error bars, standard error of the mean.

Strikingly, p190B^+/-^Neu mice developed tumors with a markedly increased latency compared with the MMTV-Neu mice (74 weeks vs. 35 weeks, *P *< 0.0001; Figure 1a), In addition, p190B haploinsufficiency significantly reduced tumor penetrance, in that 53% (8/15) of the heterozygous mice had palpable tumors as compared with 100% (20/20) of the MMTV-Neu wild-type mice (*P *< 0.001). Immunoblotting using tumor extracts confirmed that the levels of p190B were lower in the p190B^+/-^Neu tumors, as expected (Figure 1b). Haploinsufficiency for p190B also decreased tumor multiplicity (Figure 1c). To determine whether ErbB2 signaling was changed in the p190B^+/-^Neu tumors, we examined the levels of ErbB2/Neu and ErbB3; these levels were similar between the p190B^+/+^Neu and p190B^+/-^Neu tumors (Figure 1d).

### P190B^+/+^Neu and p190B^+/-^Neu tumors have similar histology and growth, but differential signaling

**Figure 2:**
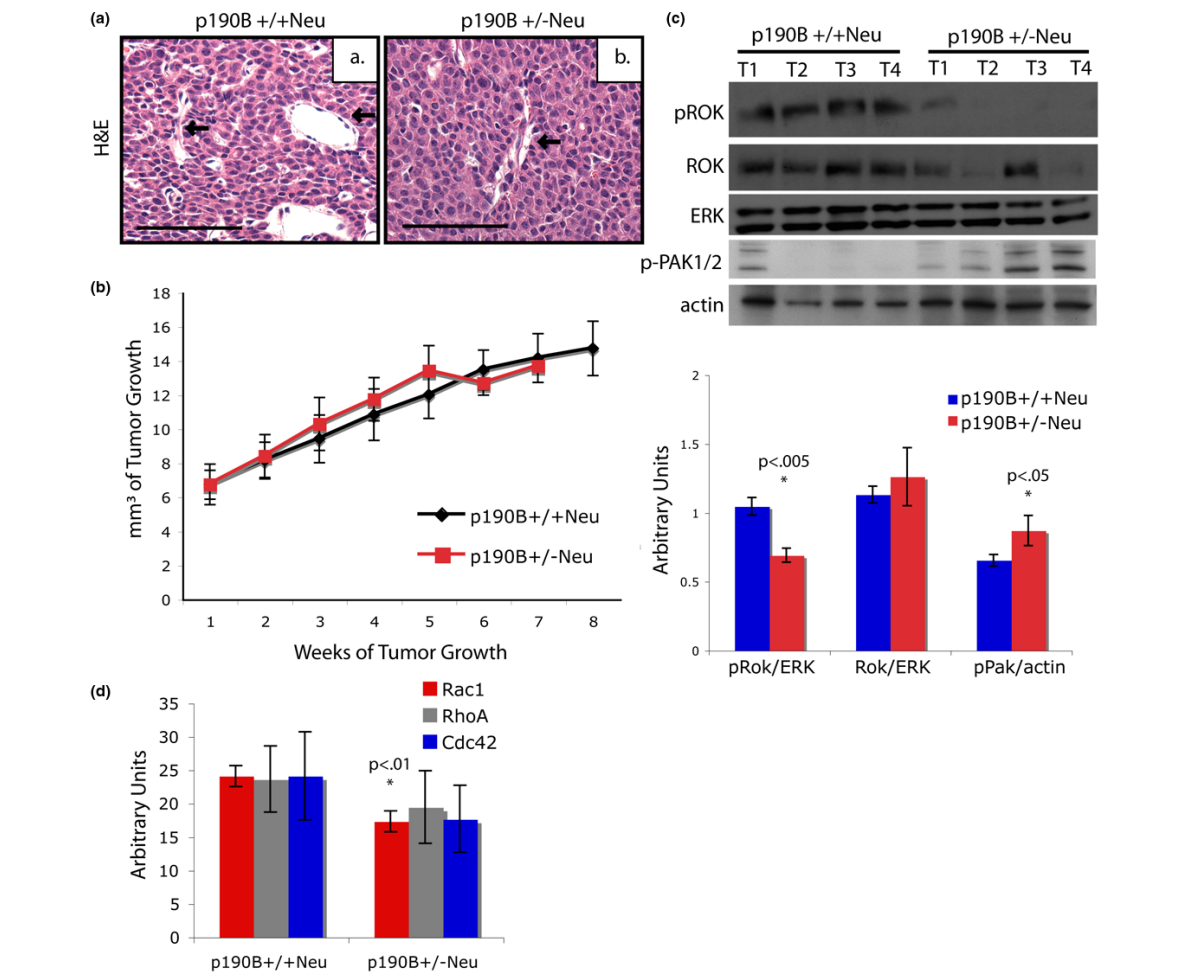
P190B^+/+^Neu tumors and p190B^+/-^Neu tumors have similar histology and growth, but differential signaling. **(a) **Histological section of p190B^+/+^Neu tumors and p190B^+/-^Neu tumors stained with hematoxylin and eosin (H&E). Arrows point to vasculature. Scale bar = 50 μm. **(b) **Growth curve of p190B^+/+^Neu tumors and p190B^+/-^Neu tumors. No statistical difference. **(c) **Immunoblotting for phosphorylated Rho kinase (pROK), Rho kinase (ROK), phosphorylated p21-activated kinase (p-PAK)1/2, and total ERK and actin as loading controls in four independent tumors from each indicated genotype. Quantification of western blots is graphed. **(d) **Quantification of active GTPase levels in tumor tissue. Analysis was carried out using Image J software. Error bars, standard error of the mean.

Histological examination showed that, similar to wild-type MMTV-Neu mice, p190B^+/-^Neu mice formed adenocarcinomas with grossly similar histopathologies. There appeared to be, however, fewer large blood vessels in the p190B heterozygous tumors as compared with the wild-type tumors (Figure 2a). The p190B^+/+^Neu and p190B^+/-^Neu tumors had similar growth patterns after initial palpation (Figure 2b). Examination of bromodeoxyuridine incorporation and cleaved caspase 3 as markers of S-phase and apoptosis, respectively, revealed no differences between the p190B^+/-^Neu tumors and the p190B^+/+^Neu tumors (data not shown).

Western blot analysis of downstream effectors of Rho, however, revealed that p190B^+/+^Neu tumors had remarkably different Rho signaling patterns as compared with the p190B^+/-^Neu tumors (Figure 2c). While p190B^+/+^Neu tumors had very little tumor-to-tumor variation, the p190B^+/-^Neu tumors exhibited variable expression of several of the downstream signaling molecules. One notable exception was that a consistent decrease in phosphorylated ROK was detected in the p190B^+/-^Neu tumors. Some of the heterozygous tumors also exhibited a decrease in total ROK. Interestingly, the levels of PAK1/2 were elevated in the p190B^+/-^Neu tumors. We also examined the levels of active Rac1, RhoA, and Cdc42. Rac1 was significantly decreased in the heterozygous tumors while RhoA and Cdc42 were unaltered (Figure 2d). These data show that heterozygosity for p190B does not affect tumor histology or growth, but does affect Rho signaling pathways.

### Haploinsufficiency for p190B blocks preneoplastic progression by reducing angiogenesis

**Figure 3:**
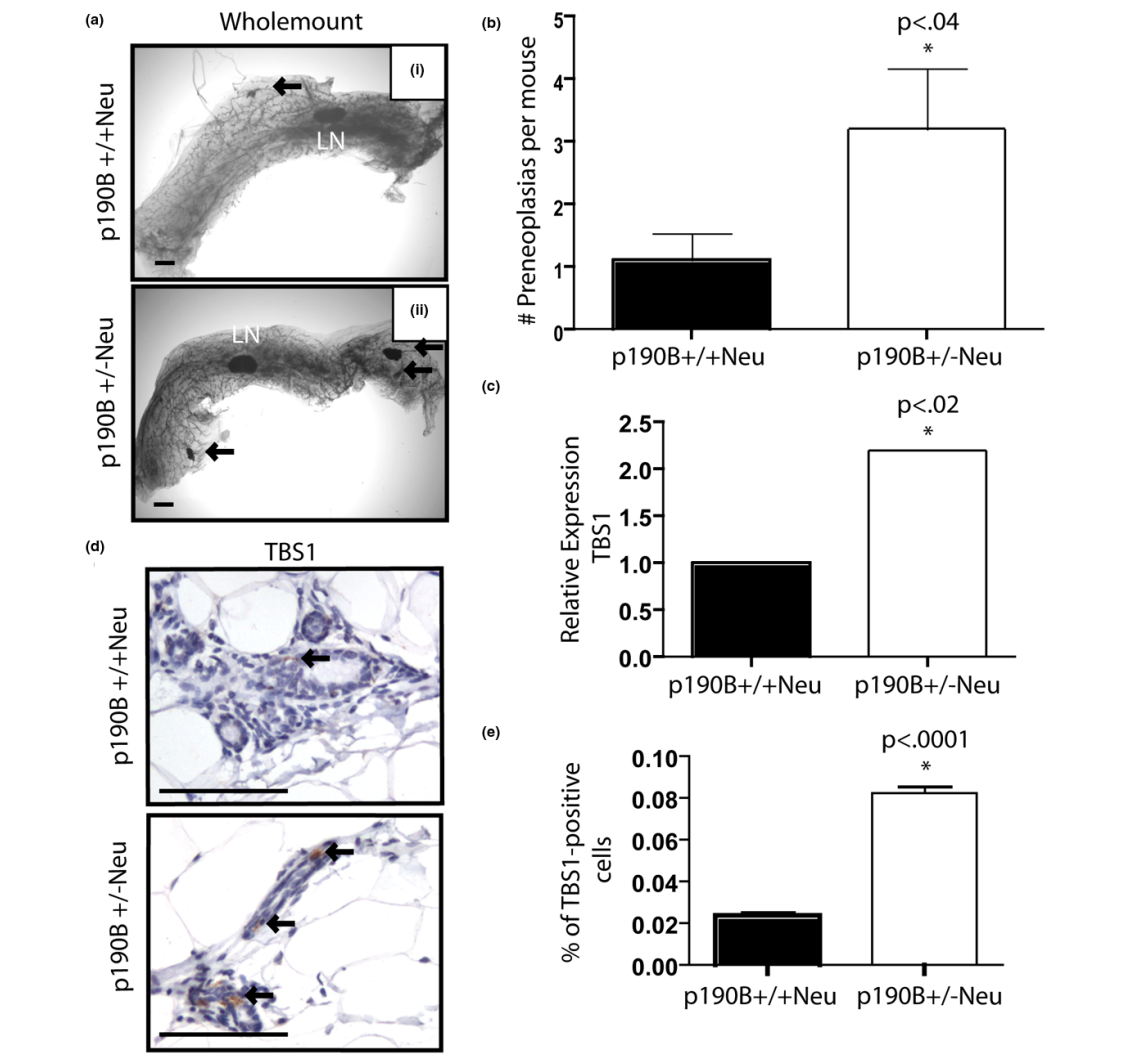
Haploinsufficiency for p190B blocks preneoplastic progression by reducing angiogenesis. **(a) **Mammary tissues from tumor burden mice with the indicated genotypes were examined by whole-mount analyses. Arrows, preneoplasias detected. LN, lymph node. Scale bar = 500 μm. **(b) **Number of preneoplastic lesions in one Number 4 inguinal gland per tumor-burden mouse was quantified. Student's *t *test showed significant difference between groups (*P *< 0.04). **(c) **Gene expression qRT-PCR data from Superarray angiogenesis array. Array analysis revealed a twofold increase in thrombospondin-1 (TBS-1) in the p190B^+/-^Neu mammary glands of adult mice 8 to 12 weeks of age. **(d) **Immunohistochemistry to detect TBS-1 protein demonstrated that TBS-1 expression is localized to the mammary epithelium and is increased in p190B^+/-^Neu mammary glands (upper) as compared with wild-type glands (lower). Images shown are at 400× magnification. **(e) **Quantification of TBS-1 immunostaining showed that TBS-1 protein is increased fourfold in p190B^+/-^Neu mammary glands. Error bars, standard error of the mean.

To determine how p190B haploinsufficiency was inhibiting tumor progression we examined using whole-mount analysis the remaining mammary glands from mice that had developed tumors. These glands exhibited many abnormalities, including enlarged ducts, alveolar-like development, and preneoplastic lesions (Figure 3a). The number of preneoplastic lesions in one number 4 inguinal gland from each mouse at the time of sacrifice for tumor burden was quantified. This analysis revealed that the p190B^+/-^Neu mice had threefold more lesions than the p190B^+/+^Neu mice (Figure 3b). The p190B^+/+^Neu lesions were more proliferative than the p190B^+/-^Neu lesions by bromodeoxyuridine incorporation, but no differences in apoptosis were detected (data not shown).

Angiogenesis is a key step in the progression of preneoplastic lesions (1 to 2 mm in size) to overt tumors [22]. We therefore considered the possibility that a block in the angiogenic switch may be one potential mechanism by which haploinsufficiency for p190B resulted in an increased number of preneoplastic lesions, but fewer tumors. To examine the gene expression of potential mediators of the angiogenic switch, mRNA isolated from 8-week-old to 12-week-old p190B^+/-^Neu and p190B^+/-^Neu virgin mammary glands was analyzed using an angiogenesis Superarray platform. While few significant changes in expression of these genes were detected, we observed a twofold increase in the mRNA expression of TBS-1 (Figure 3c and Additional data file 1), a potent angiogenic inhibitor produced by stromal fibroblasts, endothelial cells and immune cells. Furthermore, quantification of immunostaining to detect TBS-1 protein in mammary gland sections demonstrated that expression of TBS-1 was elevated twofold in the p190-B^+/-^Neu glands as compared with the wild-type glands (Figure 3d, e). These data suggest that p190B haploinsufficiency delays preneoplastic progression through inhibition of the angiogenic switch.

### Loss of p190B results in decreased tumor vasculature and fewer lung metastases

**Figure 4:**
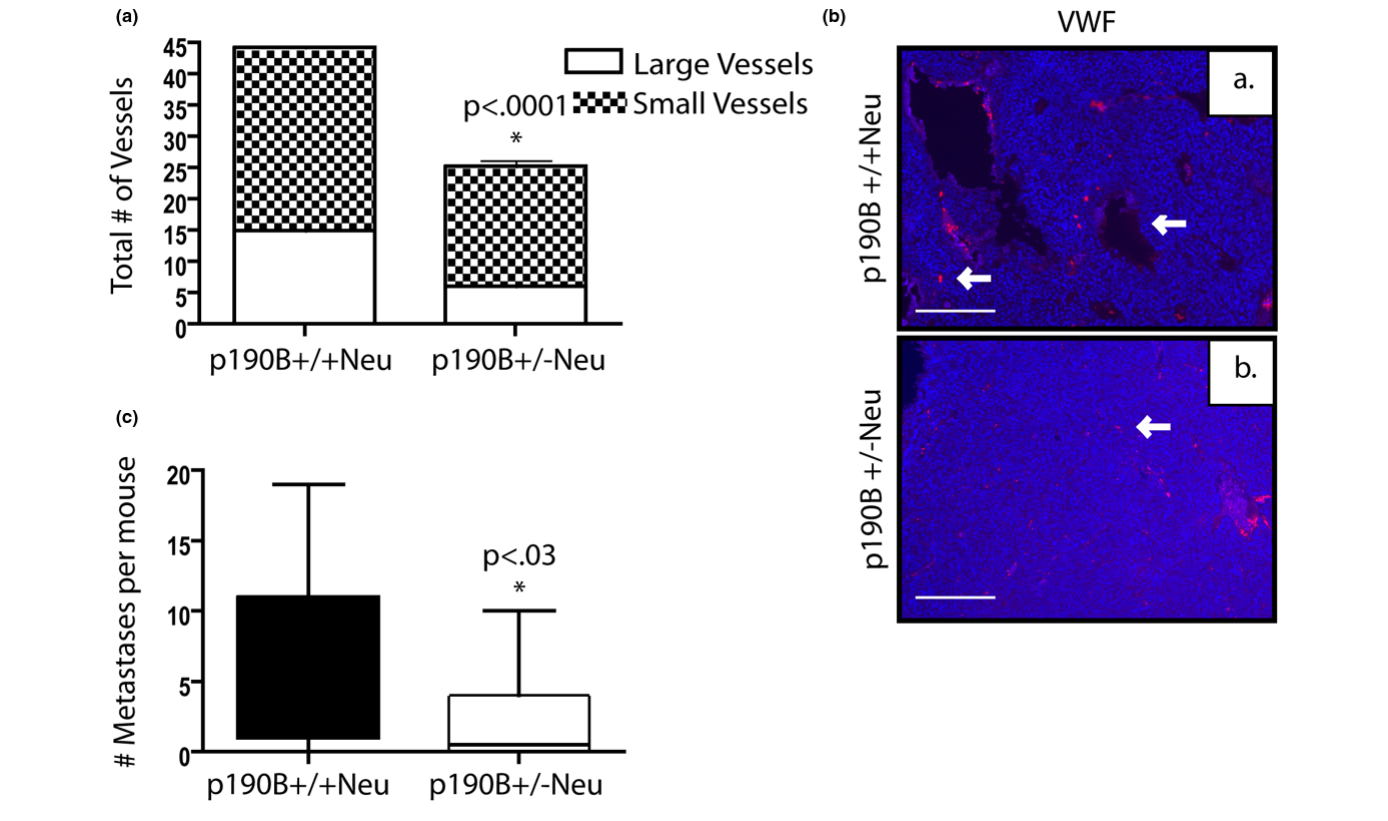
Loss of p190B results in decreased tumor vasculature and fewer lung metastases. **(a) **Number of vessels per 100× section of tumor stained with von Willebrand factor (VWF) was quantified. Student's *t *test shows a significant difference between groups (*P *< 0.0001). **(b) **Representative pictures of VWF immunofluorescence (red) and dapi (blue) in the two different genotypes as indicated. Arrows mark blood vessels. Scale bar = 100 μm. **(c) **Number of metastases per mouse at tumor burden, as determined by counting metastatic nodules in serial sections of the entire lung. Student's *t *test showed a significant decrease in p190B^+/-^Neu tumor mice (*P *< 0.03). Error bars, standard error of the mean.

To determine whether this inhibition of angiogenesis is also present in p190B^+/-^Neu tumors, we assessed the number of vessels using immunostaining for an endothelial specific marker (VWF) (Figure 4b). This analysis showed a twofold decrease in the number of vessels in the p190B^+/-^Neu tumors as compared with the p190B^+/+^Neu tumors (Figure 4a). We also examined the lungs of p190B^+/-^Neu and p190B^+/+^Neu mice with similar tumor burdens to determine whether p190B haploinsufficiency affected metastasis. The percentage of mice with metastases was not significantly different between the two genotypes due to the limited number of p190B heterozygous mice that exhibited primary tumors. The p190B^+/-^Neu mice developed fewer metastatic nodules than the p190B^+/+^Neu mice, however, despite the fact that they had similar primary tumor burdens (Figure 4c). These results suggest that haploinsufficiency for p190B inhibits tumor angiogenesis and ensuing metastatic progression of the p190B^+/-^Neu tumors.

### Angiogenesis defect in p190B^+/-^Neu tumors is rescued by wild-type stroma and growth of wild-type tumors is inhibited by heterozygous stroma

**Figure 5:**
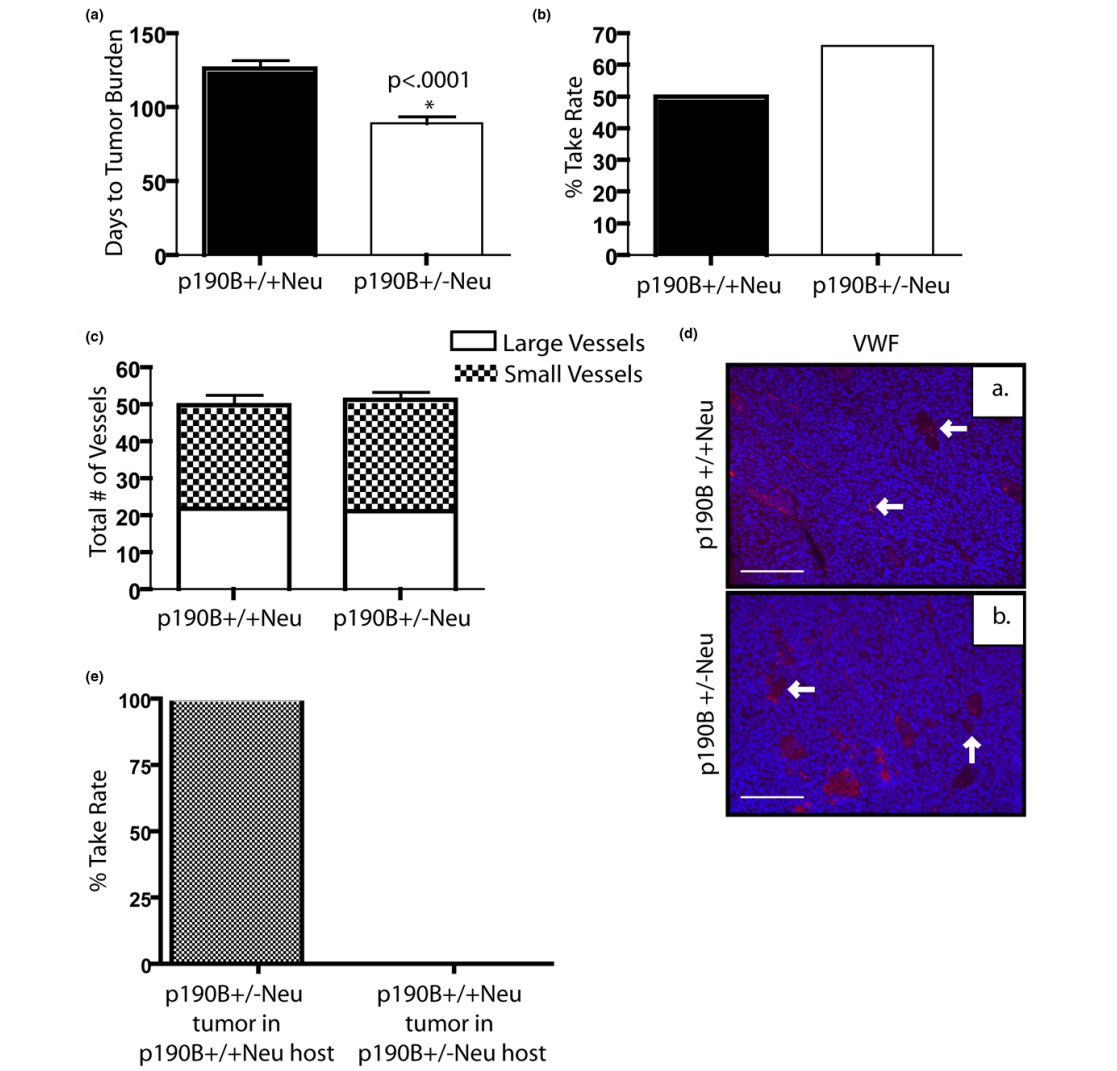
Angiogenesis defect in p190B^+/-^Neu tumors is rescued by wild-type stroma. **(a) **Number of days to tumor burden following transplantation of a designated genotype tumor piece into the cleared fatpad of a SCID/Beige mouse. Decreased time to tumor burden was statistically significant in p190B^+/-^Neu tumor transplants. **(b) **Percentage take rate of p190B^+/+^Neu transplants and p190B^+/-^Neu transplants. No statistical difference was seen between genotypes. **(c) **Number of vessels per 100× section of tumor stained with von Willebrand factor (VWF) was quantified. No statistical difference between genotypes was detected. **(d) **Representative pictures of VWF immunofluorescence (red) and dapi (blue) in the two different genotypes as indicated. Arrows mark blood vessels. Scale bar = 100 μm. **(e) **Percentage take rate of p190B^+/-^Neu tumors into a p190B^+/+^Neu host and of p190B^+/+^Neu tumors into a p190B^+/-^Neu host. The p190B^+/-^Neu tumors had a 100% take rate, while the p190B^+/+^Neu tumors had a 0% take rate. Statistically significant difference *P *< 0.0001. Error bars, standard error of the mean.

To determine the relative contribution of the epithelium and the stroma to the tumor progression process, tumor pieces from p190B^+/+^Neu tumors and from p190B^+/-^Neu tumors were transplanted into cleared fatpads of SCID/beige mice. Interestingly, the p190B^+/-^Neu tumor transplants grew faster than the p190B^+/+^Neu tumors, with a mean time to tumor burden of 89 days compared with 126 days, respectively (*P *< 0.0001; Figure 5a). The transplantation take rates were similar between the two groups (Figure 5b), with no statistical difference determined by the Fisher exact test.

We next analyzed the transplanted tumors to determine whether the vascular defect seen in the original tumors persisted in the transplants. For this analysis, the tumors were stained with VWF and the vessels were counted. Interestingly, no significant differences in the number of large and small vessels were observed in both the p190B^+/+^Neu and p190B^+/-^Neu tumor transplants (Figure 5c, d). These data indicate that the effects of p190B haploinsufficiency on MMTV-Neu tumor angiogenesis are due to defects in the vasculature and/or stroma.

Finally, we performed reciprocal transplants using p190B^+/+^Neu and p190B^+/-^Neu 5-week-old mice as the host to examine the contribution of the heterozygous stroma. The p190B^+/-^Neu tumor pieces all grew to form tumors in the p190B^+/+^Neu stroma (n = 6), while none of the p190B^+/+^Neu tumor pieces grew to form tumors in the presence of the p190B^+/-^Neu stroma (n = 6). These data further support p190B haploinsufficiency in the vasculature and/or stroma as being responsible for the tumor growth potential.

## Discussion

In the present article we show that p190B RhoGAP, a gene that is essential for mammary gland development, plays a critical role in MMTV-Neu mammary tumor progression. Haploinsufficiency for p190B increases tumor-free survival, reduces tumor penetrance, and decreases tumor multiplicity. The level of Neu transgene expression is unaltered in the p190B^+/-^Neu normal mammary tissue and tumors, and thus downregulation of transgene expression is not responsible for the inhibition of tumor progression. ErbB3 expression was also similar between the two groups, suggesting that p190B deficiency does not inhibit Neu-induced tumorigenesis by altering the epidermal growth factor receptor signaling axis that promotes tumor formation in this model [23]. Interestingly, p190B heterozygosity does not inhibit the growth rate of established tumors. Furthermore, we found in a few of the p190B^+/-^Neu tumors that p190B expression levels were comparable with the levels detected in the p190B^+/+^Neu tumors (data not shown).

It has been reported that GAPs and related G proteins are upregulated in MMTV-Neu tumors [19], and our results confirm that this signaling network plays an important role in MMTV-Neu tumor progression. Based on previous studies using p190B-deficient mouse embryonic fibroblasts [12], we anticipated that reduced p190B expression *in vivo *would increase Rho GTPase activities as well as signaling through the downstream effectors ROK and PAK. GTPase assays to examine the levels of active RhoA, Rac1, and Cdc42, however, demonstrated that only Rac1 levels were altered in the heterozygous tumors, and they were in fact decreased. In addition, ROK activity - and in some cases ROK expression levels - were reduced in the p190B-deficient tumors, whereas PAK1/2 activity levels were increased. These data indicate that p190B is required for proper regulation of Rho/ROK signaling during Neu mammary tumor progression.

Rac1 and PAK activities have been shown to play important roles in mammary tumorigenesis [24,25], and thus PAK1/2 activities may be elevated in the p190B-deficient mice to compensate for the decrease in Rac1 activity. It is interesting, and perhaps not surprising, that p190B deficiency does not result in persistent upregulation of Rho GTPase activity in the tumors. Rho GTPases play critical roles in essential cellular processes such as cell cycle progression, mitosis, and cell survival, and GTPase activity is tightly controlled during these processes [26]. Downstream effectors such as PAK and ROK are also critical for these processes [27,28]. A loss of p190B resulting in persistently elevated activity of several Rho GTPases is therefore likely to have a negative impact on cell survival or proliferation. Other GAPs or guanine nucleotide dissociation inhibitors may be upregulated or the guanine nucleotide exchange factor activity may be downregulated to compensate for the loss of p190B. In some tumors, we noted that p190B levels were similar to levels in wild-type tumors, suggesting compensatory mechanisms were functioning to restore p190B levels.

Taken together, these results demonstrate that p190B deficiency results in unexpected effects on the Rho signaling axis and reveal an important role for p190B in regulating these signaling pathways during mammary tumor progression. In addition, these results highlight the importance of investigating the role of these signaling pathways in tumorigenesis in the context of the complex *in vivo *environment.

Examination of the mammary glands from the p190B^+/-^Neu tumor-burden mice indicated that there were a higher number of preneoplastic lesions. This increase probably resulted from a lack of progression, since averaging the number of preneoplastic lesions and the number of tumors showed no statistical difference in the total number of initiated lesions between the p190B^+/+^Neu mice and the p190B^+/-^Neu mice. These preneoplastic lesions were characteristic of the avascular phase of the angiogenic switch, as they were approximately 1 to 2 mm in diameter [22,29]. Interestingly, several p190B^+/-^Neu mice did not develop tumors by 18 months of age and, when their glands were examined for preneoplastic lesions, no lesions were detected (7/7 mice) (data not shown). These data suggest that p190B is important for the progression of preneoplastic lesions, but that it may also play a role in tumor initiation. Further studies in which normal mammary tissue pieces from p190B^+/-^Neu mice are transplanted into wild-type stroma and are allowed to proceed through the stochastic process of tumor formation will be required to examine preneoplastic lesions in more detail.

The possibility that p190B was important for the progression of avascular preneoplastic lesions to vascularized lesions prompted us to examine angiogenesis in the p190B^+/-^Neu mice. To characterize the potential for a vascular defect in these mice we utilized the Superarray qRT-PCR angiogenesis platform, which allowed us to examine the expression of 87 angiogenesis genes, both positive and negative regulators, including vascular endothelial growth factor, matrix metalloproteinase-2 and matrix metalloproteinase-9, hypoxia inducible factor 1 alpha, fibroblast growth factor genes, TBS-1, and transforming growth factor beta genes. Only TBS-1 was significantly increased within the adult virgin mammary glands of the p190B^+/-^Neu mice, and quantification of immunostaining for TBS-1 confirmed this increase.

Previous studies have demonstrated that TBS-1 is a component of the extracellular matrix that functions as a negative regulator of tumor vasculature, and TBS-1 expression dramatically effects mammary tumorigenesis. For example, overexpression of TBS-1 inhibited angiogenesis in transgenic mice overexpressing activated Neu, resulting in delayed tumor onset and reduced tumor penetrance [30]. Conversely, TBS-1 deficiency in the transgenic mice overexpressing activated Neu increased angiogenesis, enhanced growth, and reduced tumor latency [30].

TBS-1 has previously been linked to GTPase signaling. Ras/Rho signaling has been shown to repress TBS-1 through effects on phosphatidyl inositol-3 kinase [31], and Ras/Rho-mediated suppression of TBS-1 has been suggested as necessary to promote angiogenesis [32]. Disruption of Rho/ROK signaling may therefore account for the elevated TBS-1 expression that was detected in the p190B^+/-^Neu mice. Increased expression of this potent angiogenic inhibitor may be responsible, in part, for the dramatic inhibition of tumorigenesis that occurred in the p190B^+/-^Neu mice.

Consistent with the increased TBS-1 expression in p190B^+/-^Neu mammary glands, p190B haploinsufficiency also resulted in decreased angiogenesis in the tumors. This persistent vascular defect may be responsible for the decreased number of metastatic nodules detected in the tumor-bearing p190B heterozygous mice. Interestingly, an increased vascular density has been correlated with poor breast cancer prognosis, and TBS-1 expression was inversely correlated with malignant progression of mammary and lung carcinomas as well as melanoma [33,34].

Our transplantation experiments showed that the reduction in angiogenesis was probably due to defects in the stroma or vasculature because transplantation of p190B^+/-^Neu tumor pieces into wild-type stroma restored angiogenesis to the levels detected in wild-type tumors. Conversely, wild-type tumors pieces failed to grow in the p190B-deficient stroma, suggesting that loss of p190B. These data are consistent with recent studies in which siRNA knockdown of p190B inhibited capillary tube formation in Matrigel by human umbilical vein endothelial cells [35]. Interestingly, these transplantation experiments revealed a decreased time to tumor burden in the p190B^+/-^Neu transplant tumors compared with the p190B^+/+^Neu tumors. The mechanism responsible for this difference remains to be elucidated. One potential explanation, however, may be that the cells which initiated the p190B^+/-^Neu tumors had already acquired significant genetic changes in order to develop the initial tumor in the absence of one allele of p190B, which in the presence of wild-type stroma impart a growth advantage. For example, PAK1/2 activity is elevated in the heterozygous tumors, and PAK1 hyperactivation promotes mammary tumor formation in part by activating p38MAPK signaling and proliferation [25]. Elevated PAK activity may therefore allow the tumor cells to respond more robustly to proliferative cues present in the wild-type stroma. Further studies, including microarray analysis of the original and transplanted tumors, as well as transplantation experiments in which p190B^+/-^Neu mammary epithelium is introduced into wild-type stroma and is allowed to go through the stochastic process of tumor formation, will be required to fully understand the contribution of p190B in the epithelium to tumor initiation, growth, and progression.

## Conclusions

Elevated expression of Rho GTPases has been detected in many types of cancer including breast cancer [36]. RhoA, RhoB, Rac1/Rac1b, and Cdc42 are overexpressed in breast tumors, with RhoA expression associated with advanced stages of the disease [6,37]. RhoC overexpression has been associated with inflammatory breast cancer and may be involved in tumor angiogenesis [5,7]. Our studies demonstrate that p190B, an inhibitor of the Rho GTPases, is required for Rho/ROK signaling during mammary tumorigenesis and point to a critical role for p190B in the vasculature during tumor progression. These studies highlight the importance of investigating the effects of altered Rho signaling in the context of the *in vivo *environment in order to elucidate the role of this signaling network in breast cancer. The functional role of Rho GTPases in the different subtypes of human breast cancer remains understudied, but these studies using mouse models suggest that this pathway may play an important role in tumor progression.

## Abbreviations

BSA: bovine serum albumin; GAP: GTPase activating protein; GTPases: guanine nucleotide triphosphatases; MMTV: mouse mammary tumor virus; Neu: rat homolog of ErbB2; PAK: p21-activated kinase; PCR: polymerase chain reaction; Rho: Ras homologous; ROK: Rho kinase; siRNA: small interfering RNA; TBS-1: thrombospondin-1; VWF: von Willebrand factor.

## Competing interests

The authors declare that they have no competing interests.

## Authors' contributions

BMH-S performed the majority of the experiments described in the paper, drafted the manuscript, and contributed to the intellectual development of the study. TV-G performed transplantation experiments, co-drafted the manuscript, and contributed to the study design, data analysis and interpretation. PRM quantified and interpreted the GTPase assays. VJ quantified and interpreted metastasis data. MPH assisted with transplantations, performed VWF and TBS-1 immunostaining, and quantified and interpreted these data. SGH participated in the design of the study and performed statistical analysis. JS provided p190B knockout mice and participated in the design and interpretation of the study. JMR directed the design and implementation of the study, interpreted data, and co-drafted the manuscript. All authors read and approved the final manuscript.

## Supplementary Material

Additional file 1 A Word file containing a table showing the fold changes and *P *values for the angiogenesis superarray comparing p190B^+/-^Neu mammary glands with p190B^+/+^Neu mammary glands. Click here for file
