# Supplementary material for: Haploinsufficiency for p190B RhoGAP inhibits MMTV-Neu tumor progression
Source: Breast Cancer Res. 2009 Aug 24;11(4):R61. doi: 10.1186/bcr2352 (PMC2750123; doi:10.1186/bcr2352)
Supplement: Additional file 1 — A Word file containing a table showing the fold changes and P values for the angiogenesis superarray comparing p190B+/-Neu mammary glands with p190B+/+Neu mammary glands. [file bcr2352-S1.doc]

Supplemental Table 1

Angiogenesis Superarray Primary Data comparing p190B+/-Neu mammary glands to p190B+/+Neu mammary glands including Fold change and P-value.

| **Gene** | **Fold Change** | **P-value** | **Gene** | **Fold Change** | **P-value** |
| --- | --- | --- | --- | --- | --- |
| Angpt1 | 24.59 | 0.136 | Nrp1 | 2.50 | 0.126 |
| Angpt2 | 5.99 | 0.114 | Nrp2 | 1.38 | 0.631 |
| Anpep | 6.50 | 0.106 | Pdgfa | 1.36 | 0.561 |
| Bai1 | 2.57 | 0.205 | Pecam1 | 1.03 | 0.973 |
| Ccl11 | 10.98 | 0.102 | Pgf | 1.24 | 0.494 |
| Ccl2 | 6.53 | 0.182 | Plau | -1.05 | 0.901 |
| Cdh5 | 11.50 | 0.149 | Plg | 1.43 | 0.403 |
| Col18a1 | 11.39 | 0.140 | Plxdc1 | -1.18 | 0.800 |
| Col4a3 | 12.88 | 0.105 | Ptgs1 | 7.34 | 0.245 |
| Csf3 | 11.63 | 0.164 | Serpinf1 | 4.24 | 0.105 |
| Ctgf | 10.20 | 0.194 | Smad5 | 2.03 | 0.255 |
| Cxcl1 | -1.22 | 0.669 | Sphk1 | 1.46 | 0.258 |
| Cxcl2 | 7.50 | 0.100 | Stab1 | 1.25 | 0.560 |
| Cxcl5 | 7.38 | 0.211 | Tbx1 | 1.01 | 0.979 |
| Ecgf1 | 5.54 | 0.172 | Tbx4 | 1.29 | 0.681 |
| Edg1 | -8.51 | 0.616 | Tek | -1.48 | 0.536 |
| Efna1 | 6.70 | 0.130 | Tgfa | -1.33 | 0.420 |
| Efnb2 | 6.36 | 0.165 | Tgfb1 | -1.69 | 0.175 |
| Egf | 6.02 | 0.166 | Tgfb2 | -1.50 | 0.438 |
| Eng | 10.75 | 0.096 | Tgfb3 | -1.25 | 0.608 |
| Epas1 | 13.64 | 0.125 | Tgfbr1 | 5.91 | 0.109 |
| Ephb4 | 14.16 | 0.125 | **Tbs1** | **2.19** | **0.022** |
| Ereg | 13.03 | 0.159 | Tbs2 | -1.13 | 0.732 |
| F2 | -1.03 | 0.972 | Timp1 | -1.83 | 0.395 |
| Fgf1 | 2.66 | 0.128 | Timp2 | -1.34 | 0.449 |
| Fgf2 | 2.11 | 0.291 | Tmprss6 | 1.09 | 0.953 |
| Fgf6 | 1.24 | 0.725 | Tnf | -1.25 | 0.576 |
| Fgfr3 | 4.53 | 0.095 | Tnfaip2 | -2.05 | 0.209 |
| Figf | 5.46 | 0.149 | Tnfsf12 | -1.95 | 0.195 |
| Flt1 | 6.33 | 0.151 | Vegfa | -2.20 | 0.111 |
| Fzd5 | 5.25 | 0.150 | Vegfb | -2.27 | 0.195 |
| Gna13 | 3.83 | 0.239 | Vegfc | -1.57 | 0.201 |
| Hand2 | 1.30 | 0.485 | Gusb | 9.11 | 0.102 |
| Hgf | 5.30 | 0.235 | Hprt1 | 1.27 | 0.170 |
| Hif1a | 8.46 | 0.213 | Hsp90ab1 | -1.73 | 0.085 |
| Ifng | -1.41 | 0.550 | Gapdh | 1.00 | NaN |
| Igf1 | 3.53 | 0.092 | Actb | -1.23 | 0.235 |
| Il1b | 4.08 | 0.200 | MGDC | 1.30 | 0.485 |
| Il6 | 2.07 | 0.242 | RTC | -1.61 | 0.162 |
| Itgav | 1.97 | 0.130 | RTC | -1.76 | 0.214 |
| Itgb3 | 2.50 | 0.159 | RTC | -2.35 | 0.105 |
| Jag1 | 3.99 | 0.046 | PPC | -1.30 | 0.639 |
| Kdr | 2.01 | 0.463 | PPC | -1.22 | 0.614 |
| Lama5 | 2.76 | 0.298 | PPC | -1.12 | 0.787 |
| Lect1 | 3.84 | 0.164 |  |  |  |
| Lep | 2.73 | 0.268 |  |  |  |
| Mapk14 | 4.22 | 0.222 |  |  |  |
| Mdk | 1.12 | 0.861 |  |  |  |
| Mmp19 | 6.79 | 0.155 |  |  |  |
| Mmp2 | 2.50 | 0.101 |  |  |  |
| Mmp9 | 2.19 | 0.256 |  |  |  |
| Npr1 | 2.05 | 0.186 |  |  |  |
